# Spatio-temporal distribution of influencing factors of cardiovascular disease in the United States

**DOI:** 10.3389/fpubh.2025.1649851

**Published:** 2025-09-11

**Authors:** Wenhui Lou, Xiaoqian Zhang, Yanchun Zhang, Xiangyang Wu, Yongnan Li, Yanhua Zhang

**Affiliations:** ^1^The Second Hospital & Clinical Medical School, Lanzhou University, Lanzhou, China; ^2^Department of Cardiac Surgery, Lanzhou University Second Hospital, Lanzhou University, Lanzhou, China; ^3^Department of Surgical Intensive Care Unit, Lanzhou University Second Hospital, Lanzhou University, Lanzhou, China

**Keywords:** cardiovascular disease, US, hot spot analysis, Moran’s I, GeoDetector, Getis-Ord Gi*, GTNNWR

## Abstract

**Introduction:**

Cardiovascular disease (CVD) is a major global health issue, contributing significantly to mortality and morbidity worldwide. The American Heart Association highlights primary prevention as a crucial strategy for mitigating the burden of CVD. This research aims to identify essential CVD drivers and support primary prevention efforts.

**Methods:**

This study analyzed data on CVD incidence across 48 states in the United States (US) from 1991 to 2020, using data obtained from the Global Burden of Disease database. To investigate the spatial–temporal heterogeneity and drivers of CVD, we employed Global Moran’s *I*, hot spot analysis, GeoDetector, and Geographically Weighted Neural Network Weighted Regression (GTNNWR).

**Results:**

Global Moran’s *I* analysis revealed significant clustering (Z-score > 2.58) of CVD rates across regions. The hotspot analysis identified significant clusters in the northeastern US. Factor detection indicated that population density, ambient particulate matter pollution, diet low in fruit, diet low in whole grain, diet high in sodium, and tobacco influenced CVD incidence. In contrast, total GDP was not statistically significant (*p* > 0.05). Interaction detection demonstrated that factors did not act independently, most interactions exhibited bilinear enhancement [q(X1, X2) > max(q(X1), q(X2))].

**Conclusion:**

Our article reveals significant spatial clustering of CVD in the US, with population density, air pollution, poor dietary patterns, and smoking emerging as major contributors. The study provides important evidence for designing geographically targeted public health interventions.

## 1 Introduction

Cardiovascular disease (CVD) is a leading global public health challenge, significantly reducing life expectancy and quality of life. The GBD 2021 study confirms the substantial contribution of CVD to the global disease burden, reporting approximately 19.42 million deaths in 2021—a 57.5% increase since 1990—and an estimated 428 million disability-adjusted life years (DALYs) attributable to CVD (1). In the US, CVD is a major driver of hospitalization and mortality, imposing heavy demands on healthcare resources and socioeconomic development. Between 2019 and 2020, CVD accounted for 12% of total national health expenditures—more than any other major diagnostic group—with total direct and indirect costs reaching $422.3 billion ($254.3 billion in medical expenses and $168.0 billion in lost productivity due to premature mortality) (2).

Effective CVD control requires treatment advancements and a greater focus on primary prevention—reducing disease occurrence by controlling risk factors before onset (3). According to the GBD database (4), the incidence of CVD in the US has shown a long-term downward trend, particularly between 1995 and 2000, largely due to a series of preventive measures implemented by the US government. Population-wide primary prevention has proven effective, as even small reductions in crucial risk factors through policy interventions can significantly lower CVD incidence (5, 6). For example, following the implementation of a comprehensive smoke-free law in New York State, the incidence of acute myocardial infarction declined significantly (5). Similarly, population-wide reductions in sodium intake have been associated with lower rates of CVD and decreased healthcare expenditures (6). Such successes emphasize that population-wide interventions can effectively reduce the disease burden and improve public health outcomes, underscoring the critical need for research on primary prevention.

Primary prevention focuses on identifying high-risk factors for disease onset and mitigating them to reduce disease likelihood. Recent research has increasingly sought to understand these risk factors, including the impact of air pollution on cardiovascular health (7, 8) and the roles of lifestyle and dietary factors in disease incidence (9). However, existing studies often have several limitations:

- Most are small-scale epidemiological studies, leading to potential biases (10, 11);
- They tend to focus on single or limited risk factors, failing to address multifactorial interactions comprehensively (12);
- The spatial heterogeneity of disease distribution is not adequately considered (10–12).

Addressing these limitations requires an analytical approach that simultaneously captures spatial heterogeneity and multifactorial interactions to provide a more comprehensive understanding of CVD risk factors.

GeoDetector has become popular in spatial studies for its ability to assess spatial heterogeneity and multifactorial interactions. The factor detector evaluates each factor’s effect on CVD distribution, while the interaction detector explores interactive effects between factors (13), thereby identifying key driving factors that aid targeted interventions. GeoDetector has been employed to investigate the spatial risk of diseases, such as neural tube defects in Heshun Region, China (14), and the spatio-temporal dynamics of COVID-19 transmission (15–18), underscoring its utility as a methodological tool in epidemiological and public health research.

Recent research has increasingly employed machine learning and deep learning models to explore the spatial and temporal dynamics of CVD. For example, Kang et al. (19) proposed an explainable AI framework to analyze spatiotemporal risk factors of cardiovascular mortality in South Korea, while Dong et al. (20) utilized machine learning to investigate regional disparities in premature cardiovascular mortality across US counties. These studies underscore the growing interest in integrating AI-based methods into public health research.

However, most of these approaches rely on traditional feature importance rankings or black-box predictions, and few are specifically designed to model spatiotemporal non-stationarity—a key characteristic of epidemiological data. To address this gap, we introduce the GTNNWR model, which combines spatial weighting and spatiotemporal proximity through neural networks to capture local variations over both space and time (21).

By integrating GTNNWR with classical spatial statistics and GeoDetector, this study aims to: identify key drivers of CVD incidence across the US; assess their spatial heterogeneity; and examine interactive and spatiotemporally varying effects, providing a more comprehensive understanding of CVD determinants to inform geographically targeted prevention strategies.

## 2 Methodology

### 2.1 Data source

This study used age-standardized CVD incidence data (1991–2020) and 29 influencing factors across four domains. Data sources include the GBD database, NOAA, and Google Earth Engine. Full details are listed in Table 1.

**Table 1:** Influential factors affecting CVD rates in the US.

Factor	Data source	Collection period
cardiovascular disease incidence	Institute for Health Metrics and Evaluation. GBD Results Tool. Available from: http://ghdx.healthdata.org/gbd-results-tool. Accessed 1 Aug 2024	1991–2020
Population density, SDI, GDP, Person income, Ambient ozone pollution, Ambient particulate matter pollution, Household air pollution from solid fuels, Nitrogen dioxide pollutionDiet Low in Omega-6 Polyunsaturated Fatty Acids, Diet high in processed meat, Diet high in red meat, Diet high in sodium, Diet high in sugar-sweetened beverages, Diet high in trans fatty acids, Diet low in fiber, Diet low in fruits, Diet low in legumes, Diet low in nuts and seeds, Diet low in seafood omega-3 fatty acids, Diet low in vegetables, Diet low in whole grains, Dietary risks, High alcohol use, Low physical activity, Tobacco,	Institute for Health Metrics and Evaluation. GBD Results Tool. Available from: http://ghdx.healthdata.org/gbd-results-tool. Accessed 1 Aug 2024	1991–2020
Temperature, precipitation	National Oceanic and Atmospheric Administration. Available from: https://www.noaa.gov. Accessed 1 Aug 2024.	1991–2020
DEM	SRTMDEM 90 m. Google Earth Engine. Available from: https://code.earthengine.google.com/. Accessed 1 Aug 2024.	1991–2020
NDVI	MODIS MYD13A2. Google Earth Engine. Available from: https://code.earthengine.google.com/. Accessed 1 Aug 2024.	1991–2020

Alaska and Hawaii were excluded due to their distinct geographic and socioeconomic conditions, which violate the spatial continuity assumptions of the GeoDetector model. Their inclusion may have introduced bias into spatial pattern detection. The final dataset covered 48 contiguous US states, enabling robust assessment of spatiotemporal heterogeneity and key CVD drivers.

### 2.2 Operational definitions

To ensure clarity and consistency in terminology, this subsection provides concise definitions of key terms, models, and statistical concepts used throughout the study. Given the integration of spatial analysis, epidemiology, and machine learning, standardized definitions facilitate accurate interpretation and reproducibility.

We also summarized the operational definitions. Table 2 presents definitions grouped by category, including spatial statistical indicators, GeoDetector methodology, and components of the GTNNWR model. Additionally, Supplementary file 1 provides definitions of disease-related terms, including criteria for high or low intake of various dietary components. These definitions serve as a reference point for understanding the analytical framework and results discussed in subsequent sections.

**Table 2:** Operational definitions of key terms used in this study.

Category	Term	Definition
Disease & Database	CVD	All cardiovascular conditions included in the GBD database, such as ischemic heart disease, stroke, and hypertensive heart disease.
	GBD Database	A global health database by IHME providing standardized estimates of disease incidence, mortality, and risk factors across time and regions.
Spatial Statistics	Global Moran’s I	A measure of spatial autocorrelation (range: −1 to +1), indicating clustering of similar values across space.
	Z-score	Standardized value used to assess the significance of clustering. Z > 2.58 denotes significance at the 99% confidence level.
	Getis-Ord Gi*	A spatial statistic identifying “hot spots” and “cold spots” based on local clusters of high or low values and their neighbors.
	Hot Spot/ Cold Spot	Areas with significantly high (hot) or low (cold) CVD incidence identified via Gi* analysis.
GeoDetector Method	GeoDetector	A spatial analysis method that detects spatial stratified heterogeneity and quantifies explanatory power of independent variables.
	q-statistic	A value between 0 and 1 quantifying the explanatory power of a variable. Higher values indicate stronger influence on the dependent variable.
	Discretization	The transformation of continuous variables into categorical bins before GeoDetector analysis (e.g., equal interval, quantile, natural breaks).
Deep Learning Models	GTNNWR	A neural network model capturing spatiotemporal non-stationarity by combining spatial and temporal weighting mechanisms.
	GNNWR	A predecessor of GTNNWR, focusing only on spatial non-stationarity using neural networks.
	STPNN	A neural module within GTNNWR that computes spatiotemporal distances and generates temporal weights.
	SWNN	A neural module within GTNNWR that computes spatial weights based on geographic proximity and feature similarity.

### 2.3 Statistical analysis

**Figure 1:**
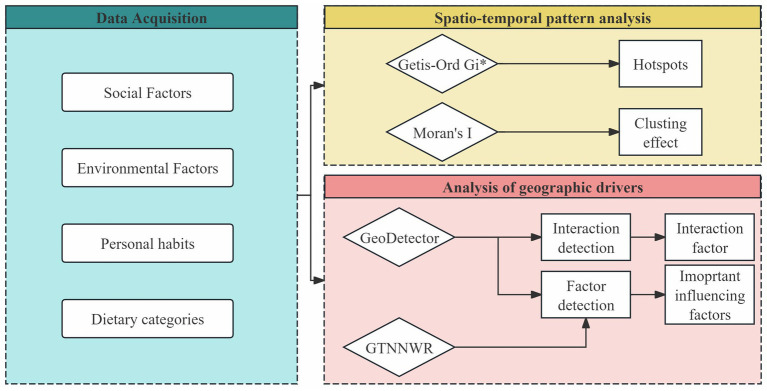
Analytical framework for exploring the spatial–temporal heterogeneity and drivers of CVD incidence.

We utilized multiple analytical methods to investigate the spatial–temporal heterogeneity and driving factors of CVD incidence. The detailed analytical process is illustrated in Figure 1, which outlines the overall workflow of the study. The analysis begins with data acquisition, where 29 influencing factors are grouped into four categories: social factors, environmental factors, personal habits, and dietary categories. These variables form the basis for subsequent analyses. Spatial clustering patterns of CVD incidence are then examined using Global Moran’s I and Getis-Ord Gi*, allowing the identification of significant aggregation effects and localized hot spots. To further explore the geographic drivers behind these patterns, we employ GeoDetector to quantify the independent and interactive explanatory power of each factor, and GTNNWR to model complex, non-stationary spatiotemporal relationships. Together, these methods provide a comprehensive framework for understanding both the spatial distribution and underlying determinants of CVD across the US. The specific analytical procedures and implementation details for each method are presented in the subsequent sections.

#### 2.3.1 Global Moran’s I

We applied Global Moran’s *I* to evaluate the overall spatial autocorrelation of CVD incidence. Spatial autocorrelation measures the degree to which values at geographically proximate locations resemble one another. A positive Moran’s *I* (values > 0) suggests that areas with high (or low) CVD incidence are near other areas with similarly high (or low) values, indicating clustering. Conversely, a negative Moran’s *I* (values < 0) implies a spatially dispersed pattern, where high and low values are interspersed. A Moran’s *I* close to zero reflects a random spatial distribution. In this study, a Z-score > 2.58 was considered statistically significant, denoting strong spatial clustering of CVD incidence.

#### 2.3.2 Getis-Ord Gi*

To identify localized clusters of high or low CVD burden, we employed the Getis-Ord Gi* statistic. Unlike Global Moran’s I, which provides an overall measure, Gi* identifies specific spatial units that contribute to clustering. For each unit, a z-score is computed based on both its value and those of its neighbors. Units with significantly high z-scores and neighboring high values are classified as “hot spots,” while those with significantly low values are labeled “cold spots.” This approach allowed us to detect regional concentrations of CVD incidence that may warrant focused public health attention.

#### 2.3.3 GeoDetector

GeoDetector was used to assess spatial stratified heterogeneity and evaluate individual factors’ independent effects and interactions. The method quantifies the explanatory power of each factor using a q-statistic, with higher values indicating stronger associations with the dependent variable. Interaction types—such as bilinear enhancement, nonlinear enhancement, or weakening—were determined by comparing the q-values of individual factors and their combinations. Specific rules for classifying interaction types are summarized in Table 3, following the standard GeoDetector framework. The GeoDetector model was implemented using the “GD” package in R 4.3.2 or higher. The input data were continuous type variables, and we discretized the data using various classification methods (equal interval, geometric interval, natural breaks, quantile) and determined the optimal method by comparing the results. The final discretization classification details are provided in Supplementary file 2.

**Table 3:** Interaction detection meaning.

Formula	Interaction
q(X1, X2) < Min(q(X1),q(X2))	Weaken, nonlinear
Min(q(X1),q(X2)) < q(X1, X2) < Max(q(X1),q(X2))	Weaken, unilateral
q(X1, X2) > Max(q(X1),q(X2))	Enhance, bilinear
q(X1, X2) = q(X1) + q(X2)	Independent
q(X1, X2) > q(X1) + q(X2)	Enhance, nonlinear

#### 2.3.4 GTNNWR

**Figure 2:**
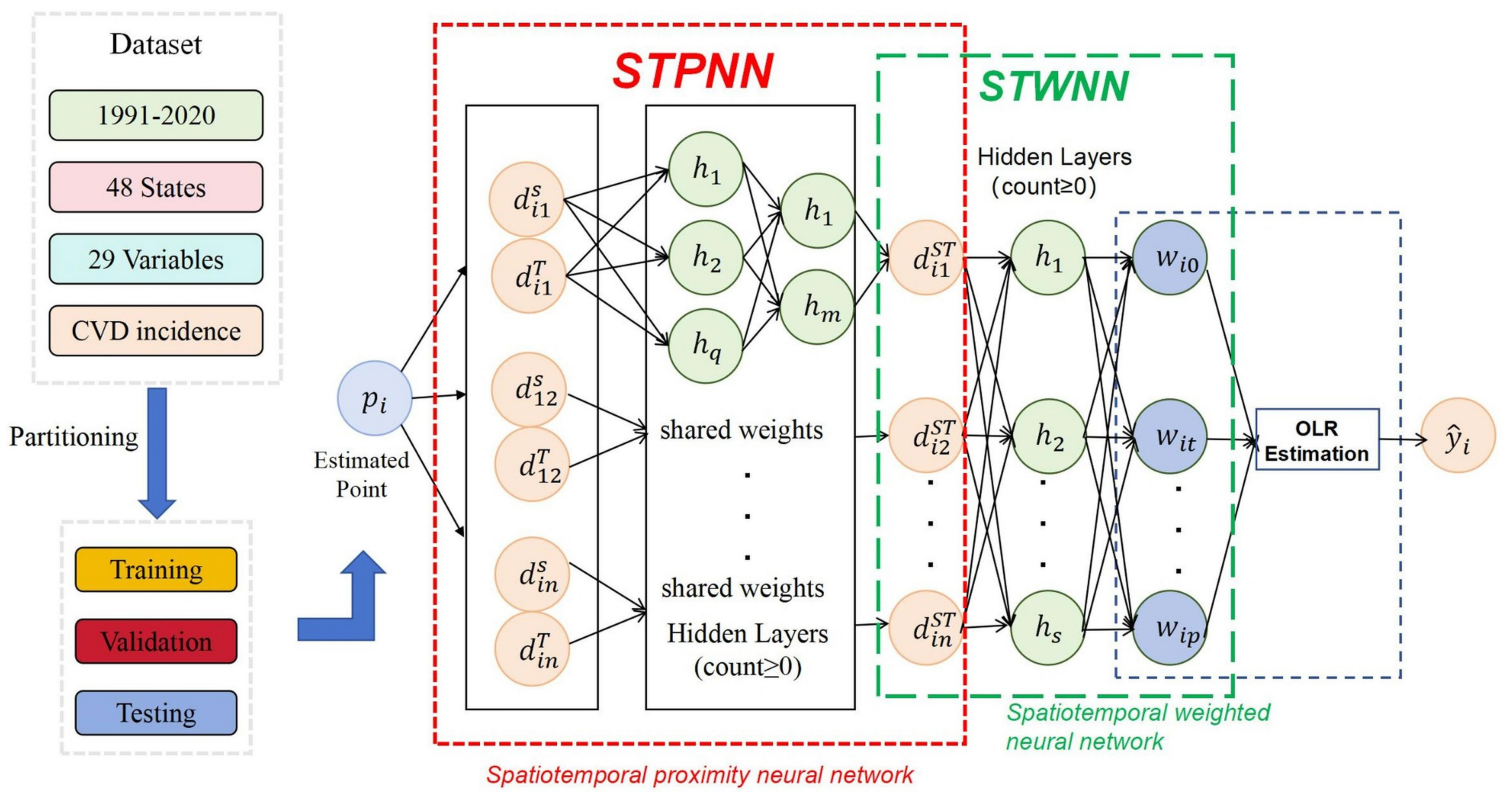
Framework of the modified GTNNWR model for CVD risk factor estimation.

Geographically Weighted Neural Network Weighted Regression (GTNNWR) is designed to model spatiotemporal non-stationary relationships, addressing variations in spatial feature relationships caused by changes in spatiotemporal structures. To tackle spatiotemporal non-stationarity, the model incorporates spatiotemporal distance into the geographically neural network weighted regression (GNNWR) framework and introduces a spatiotemporal proximity neural network (STPNN) for precise spatiotemporal distance calculations. Integrating STPNN with the spatial weight neural network (SWNN) within the GTNNWR model computes a spatiotemporal non-stationary weight matrix, enabling a more accurate representation of spatiotemporal non-stationary relationships (21). Our study utilized Python 3.11 or higher to construct the GNNWR model (https://github.com/zjuwss/gnnwr). The model’s specific parameters are detailed in Table 4. We have included a schematic diagram illustrating the working principle of GTNNWR in Figure 2. This diagram illustrates the workflow of the modified GTNNWR model applied to identify and quantify the spatiotemporal driving factors of CVD incidence across the US.

**Table 4:** Model parameter.

Parameter	Value
Architecture	[[3], [512, 256, 64]]
Drop Out	0.4
Optimizer	Adadelta
Optimizer Scheduler	MultiStepLR
Scheduler Milestones	[1,000,2000,3,000,4,000]
Scheduler Gamma	0.8
Epochs	15,000
Log Interval	1,000

First, for each estimation point (pᵢ; representing a specific state and year), the dataset is duplicated to construct a comparison set including all training samples. The spatiotemporal distances—including spatial distance (dˢ), temporal distance (dᵗ), and their combined effect (dˢᵗ)—are calculated between pᵢ and each training sample. These distances are then passed through a Spatiotemporal Specific Neural Network, which learns a nonlinear transformation to generate instance-specific weights that reflect the proximity of each training sample to the estimated point in both space and time. These learned weights capture the heterogeneity and non-stationarity of relationships between risk factors and CVD incidence. The weighted distances are fed into the Weighted OLR (Ordinary Linear Regression) component. Here, instance-specific weights are used to perform localized regression, where the influence of each explanatory variable (e.g., PM₂.₅ concentration, dietary sodium intake, smoking prevalence, and population density) on CVD incidence is estimated individually for each location-time pair. This allows the model to derive adaptive and interpretable coefficients (βᵢ) that vary across both space and time. The final output is an estimated value (ŷᵢ), representing the predicted CVD incidence for the point pᵢ, which fully accounts for the dynamic influence of risk factors in different spatiotemporal contexts.

We adopted a hold-out validation strategy to evaluate the GTNNWR model’s performance. Specifically, the dataset was randomly split into 75% training, 10% validation, and 15% testing sets using a fixed random seed (seed = 48) to ensure reproducibility. The model was trained on the training set, its hyperparameters were tuned and early stopping monitored on the validation set, and final performance was assessed on the independent test set. We did not use k-fold cross-validation due to the high computational cost of graph construction and spatiotemporal weighting in the GTNNWR architecture.

The GTNNWR model was trained using the Normalized Mean Squared Error (NMSE) loss function. NMSE penalizes large deviations more heavily and is well suited for continuous regression tasks such as spatial prediction of CVD incidence. This choice ensures sensitivity to outliers and enables stable gradient behavior during optimization.

This architecture allows the model to overcome the limitations of global regression by learning localized patterns, which is particularly important in understanding how CVD determinants vary across different states and periods in response to changing environments and public health policies.

## 3 Results

### 3.1 Spatial distribution of CVD in the US

**Figure 3:**
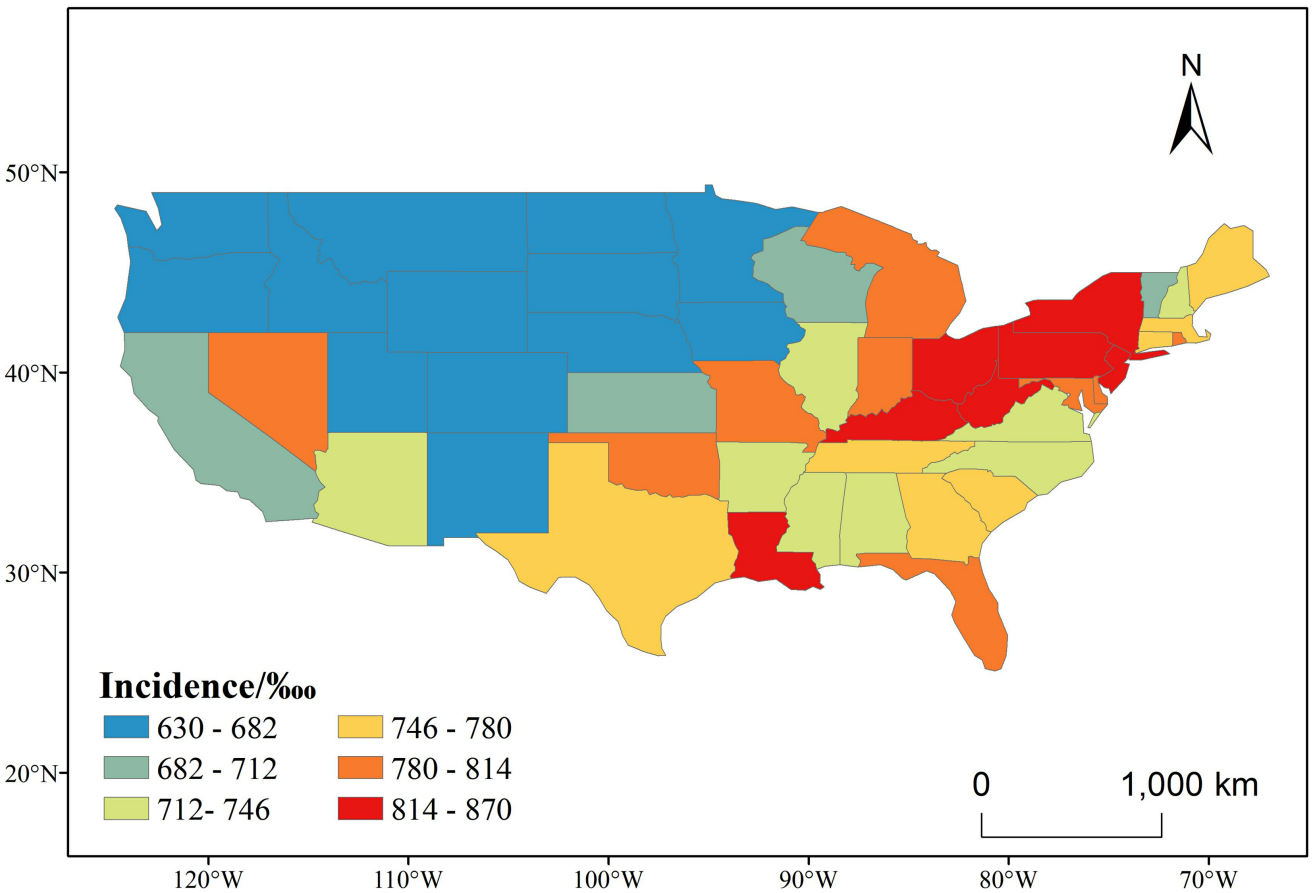
Spatial distribution of CVD incidence (1991–2020).

Figure 3 shows the cumulative incidence of CVD over the full study period (1991–2020), further highlighting persistent regional disparities. States in the Midwest, South, and Northeast—including Ohio, Pennsylvania, West Virginia, and Mississippi—exhibited the highest cumulative incidence levels (>814‱). In contrast, much of the West and Upper Midwest, including states such as Colorado, Utah, and North Dakota, maintained comparatively low rates (<682‱). This long-term pattern suggests a stable east–west gradient in CVD burden, potentially shaped by persistent differences in socioeconomic status, health behaviors, access to care, and environmental exposures.

**Figure 4:**
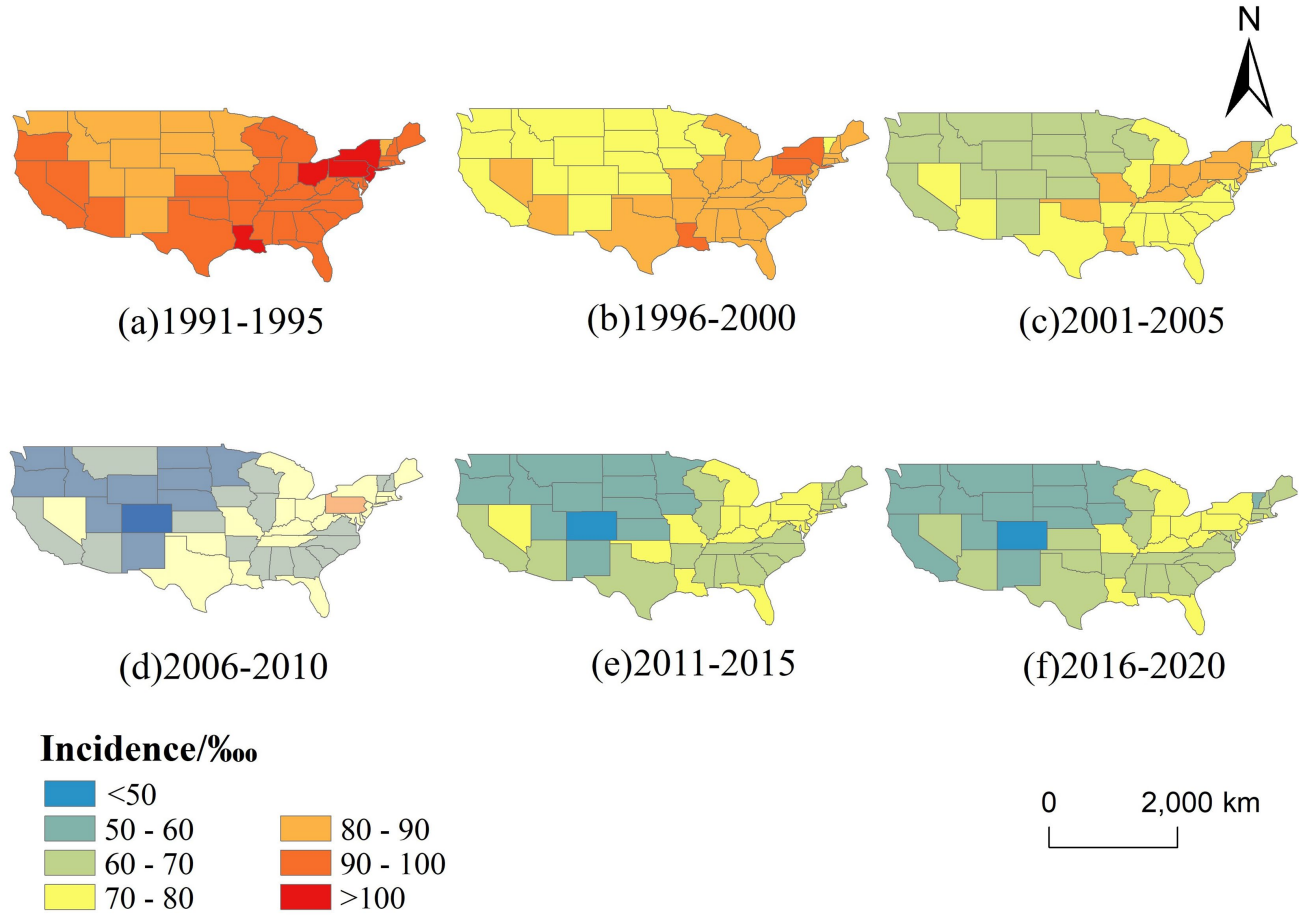
Temporal changes in the spatial distribution of CVD incidence in the US (1991–2020, 5-year intervals).

Figure 4 illustrates the spatial distribution of CVD incidence across the contiguous US from 1991 to 2020, segmented into six five-year intervals. A clear temporal trend of geographic redistribution is observed. In the early 1990s (1991–1995), high-incidence areas (≥90‱)were concentrated in the eastern and southeastern states, particularly in New York, Kentucky, and Louisiana. Over time, these high-risk areas gradually shrank, and by 2016–2020, much of the country—especially the western and northern states—fell into the lower incidence categories (<70‱), suggesting an overall decline in disease burden and a weakening of spatial clustering.

Together, these spatial patterns underscore the need for regionally targeted interventions. While national-level prevention strategies have contributed to a general decline in CVD incidence, the persistence of high-burden clusters in specific states highlights the importance of localized public health responses tailored to regional characteristics.

### 3.2 Spatial analysis of CVD incidence using Moran’s I and Getis-Ord Gi*

**Figure 5:**
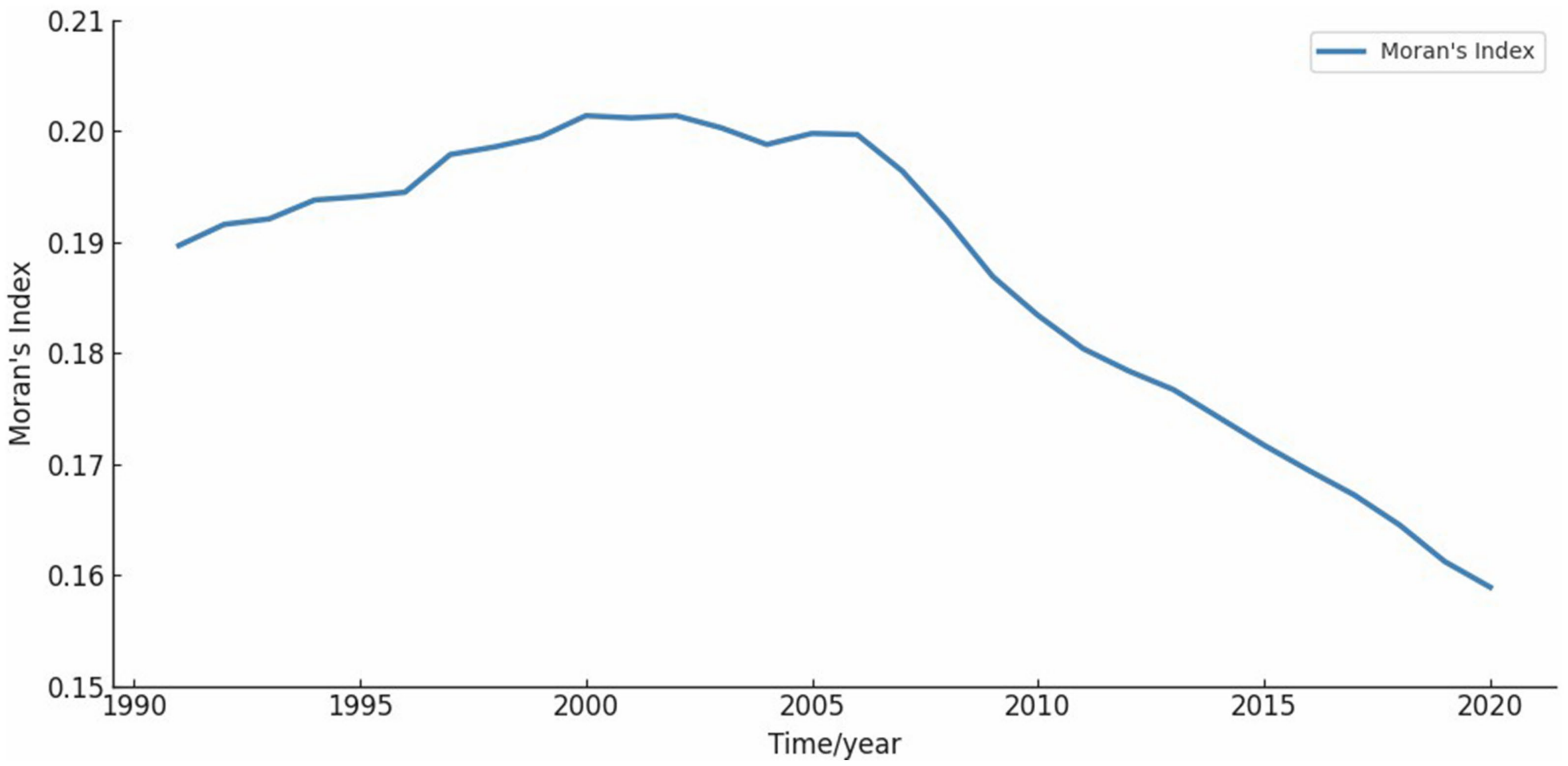
Moran’s *I* on a one-year scale. The value of Moran’s *I* show an n-shaped curve, which has gradually decreased since 2002.

Global Moran’s *I* was used to analyze the spatial agglomeration characteristics of CVD incidence data from 1991 to 2020, grouped by five-year intervals. Moran’s *I* results demonstrated significant spatial clustering across all groups (Z > 2.58), rejecting the null hypothesis (H_0_) of random spatial distribution for CVD incidence (Table 5). The annual Moran’s *I* values followed an n-shaped trend, peaking in 2002 (Figure 5).

**Table 5:** Moran’s I on a five-year scale.

Year	Moran index	Z
1991–1995	0.1926	5.2160
1996–2000	0.1995	5.3771
2001–2005	0.2001	5.3858
2006–2010	0.1920	5.1798
2011–2015	0.1764	4.8013
2016–2020	0.1642	4.5124

**Figure 6:**
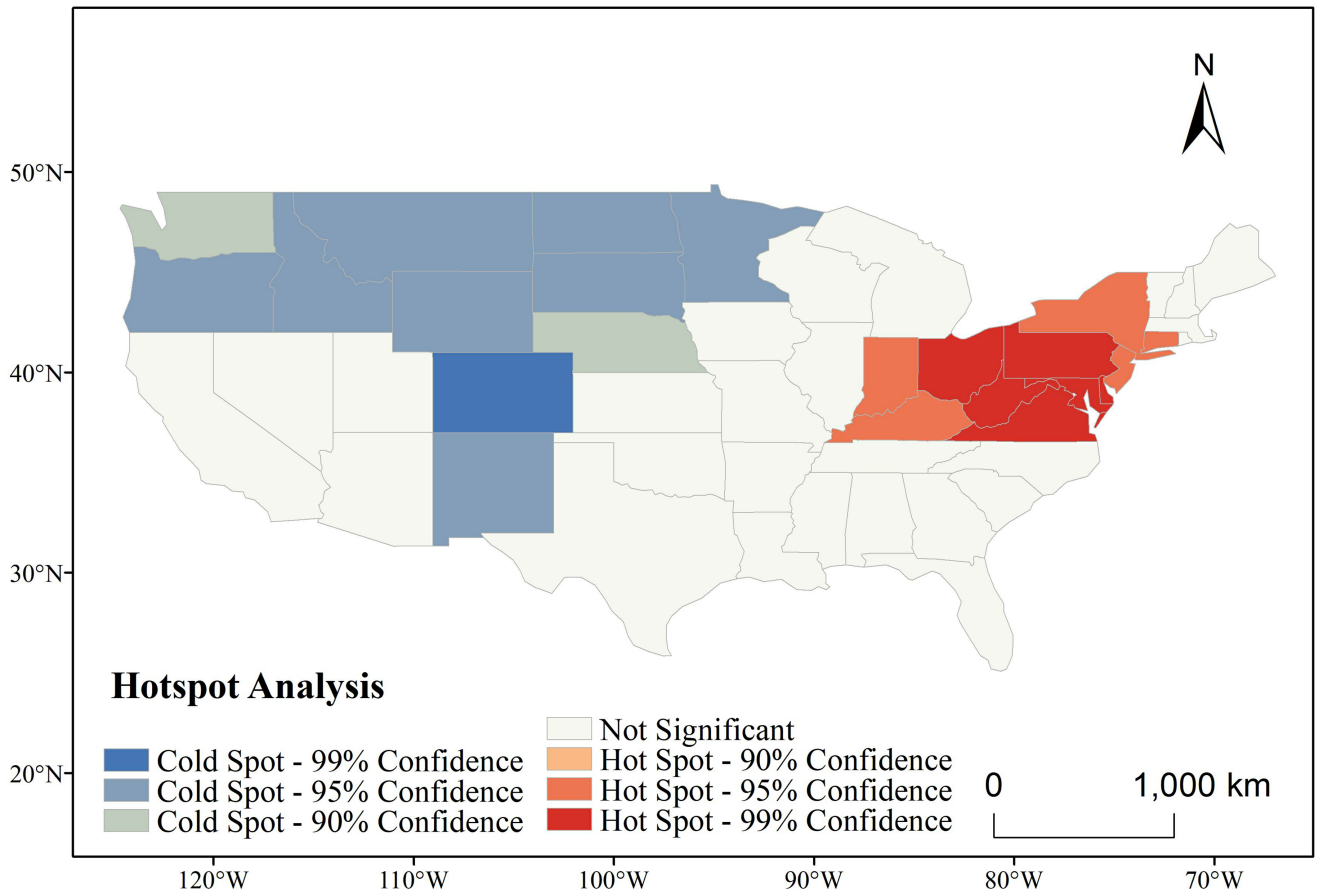
Hotspot analysis of CVD incidence in the US (1991–2020).

Figure 6 presents the spatial clustering of CVD incidence across the contiguous US, as identified by Getis-Ord Gi* analysis. Over the entire study period (1991–2020), a clear east–west contrast is evident. Statistically significant hot spots (areas with consistently high CVD incidence) were concentrated in the northeastern and central Appalachian states, including West Virginia, Ohio, Pennsylvania, Kentucky, and Maryland—many of which reached the 99% confidence level. In contrast, cold spots (areas with significantly low incidence) were primarily located in the northwestern and central mountain states, such as Colorado, Montana, and North Dakota.

**Figure 7:**
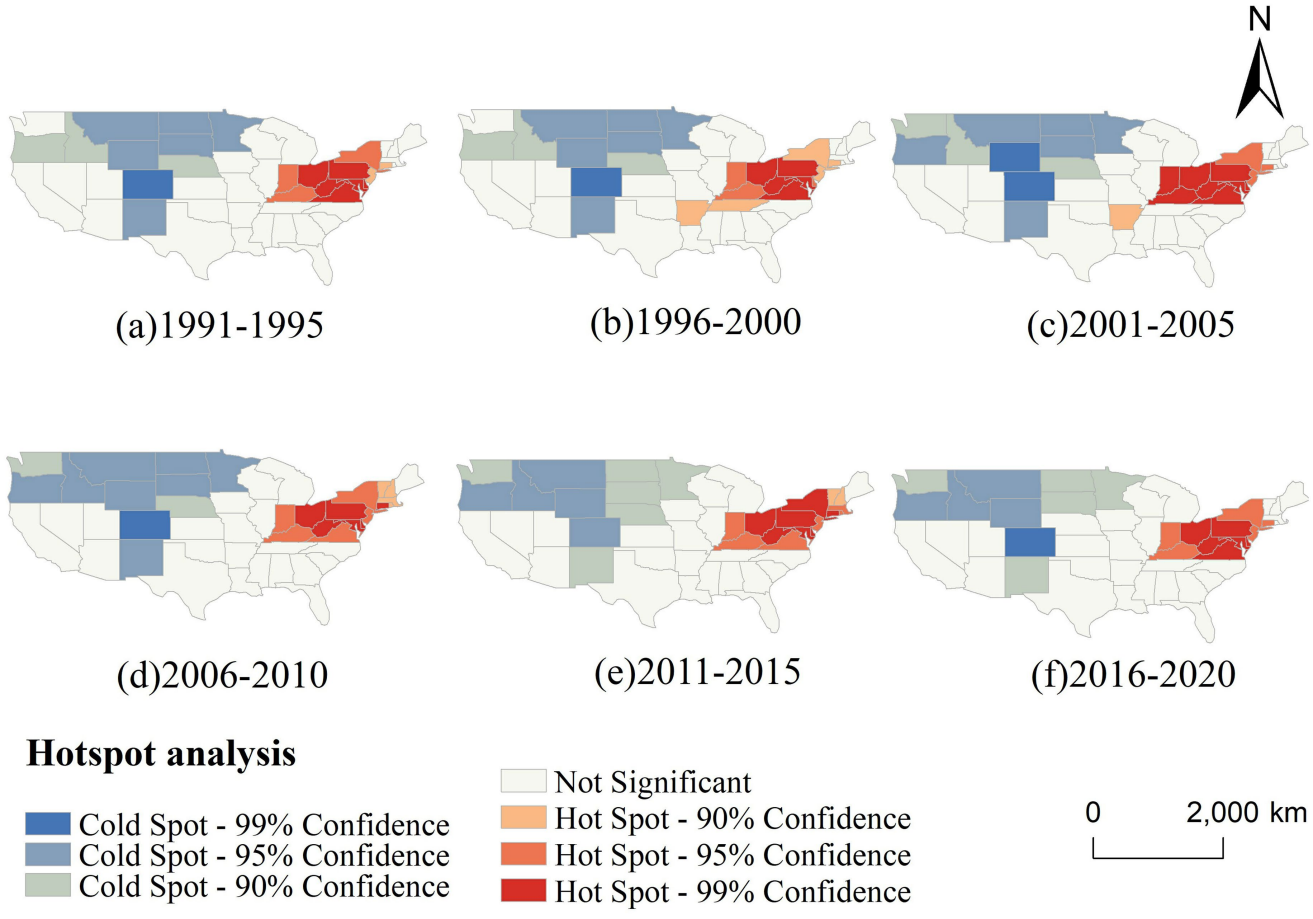
Temporal changes in the hotspot analysis of CVD incidence in the US (1991–2020, 5-year intervals).

Figure 7 further illustrates the temporal evolution of CVD hot and cold spots across six five-year intervals. Throughout the entire period, hot spots remained remarkably stable, persistently occupying the northeastern and Midwestern regions. Notably, the core cluster—comprising Ohio, West Virginia, and Pennsylvania—maintained 99% confidence levels across all time windows, indicating long-term structural determinants of elevated risk in these regions. Meanwhile, cold spots in the western US, particularly in Colorado and surrounding states, also remained stable, suggesting persistent environmental or behavioral protective factors.

The spatial persistence of these clusters suggests entrenched geographic health disparities. The northeastern hot spot belt may be associated with a combination of aging populations, socioeconomic disadvantage, poor dietary habits, and high smoking prevalence, while the cold spots in the western and northern states may reflect healthier lifestyles, better access to preventive care, or more favorable environmental exposures.

These results underscore the importance of geographically targeted public health strategies. Interventions should prioritize high-incidence areas with tailored approaches that account for persistent local risk profiles.

### 3.3 Identification of key driving factors of CVD incidence

**Figure 8:**
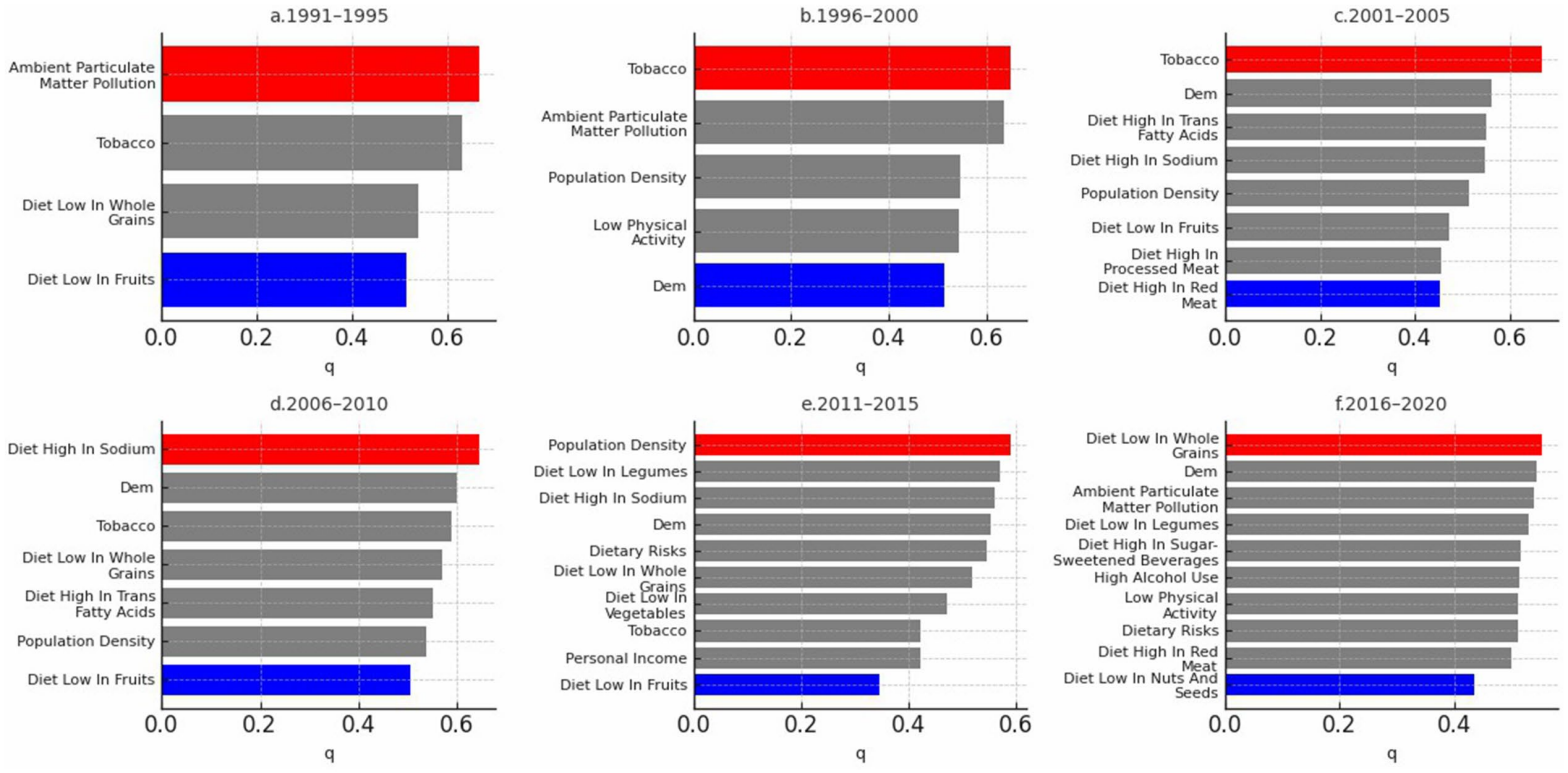
GeoDetector single-factor analysis of CVD incidence in the US (1991–2020).

Factor detection analysis using the GeoDetector model identified significant driving factors for CVD incidence (*p* < 0.05; Supplementary file 3). Population density, ambient particulate matter pollution, diet low in fruits and diet low in whole grain, diet high in sodium, and tobacco, exhibited high explanatory power across multiple periods. In contrast, economic factors such as GDP did not significantly influence CVD incidence, and their effects varied over time. The q-value in GeoDetector quantifies the explanatory power of each factor, with values closer to 1 indicating greater explanatory strength. Based on the q-values, with higher values indicating greater importance, we used red to highlight the most influential factor and blue to indicate the least influential factor in each five-year period. As shown in Figure 8, population density, ambient particulate matter pollution, and dietary risks—such as low intake of fruits and whole grains—were consistently among the most influential factors across multiple periods. In contrast, macroeconomic indicators such as GDP and environmental variables like precipitation were often among the least influential. This pattern suggests a stable and persistent influence of behavioral and environmental risks on CVD incidence, while socioeconomic factors showed weaker or inconsistent associations over time.

**Figure 9:**
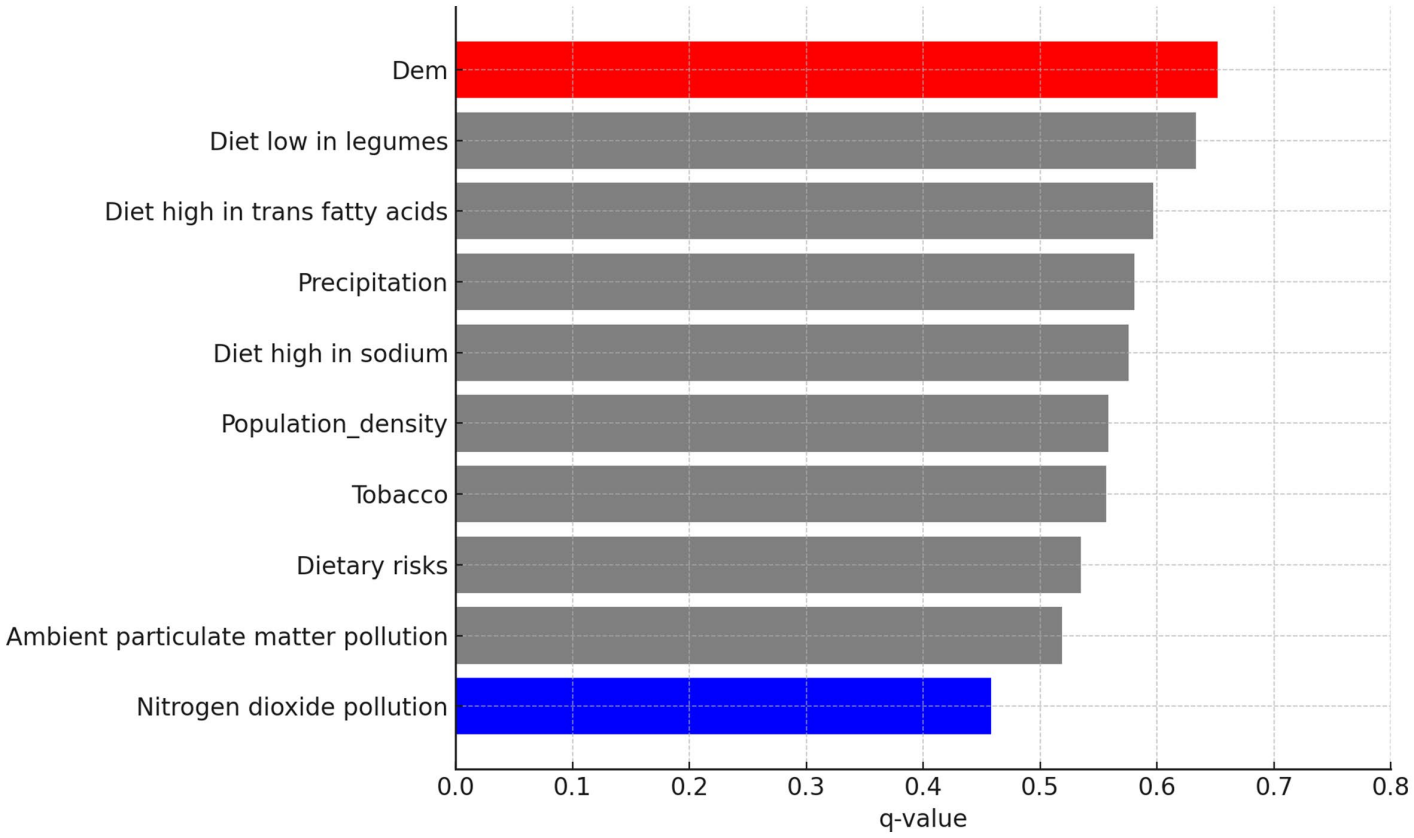
GeoDetector single-factor analysis results across the entire period.

In addition to the 5-year interval analysis, the overall factor detection results for the full 1991–2020 period are shown in Figure 9. The ranking pattern remains broadly consistent, with population density, trans fatty acid intake, and sodium-rich diets among the strongest explanatory variables. Notably, DEM (elevation) emerged as the most influential factor overall, while nitrogen dioxide pollution showed the weakest explanatory power. This suggests that over longer time spans, objective environmental conditions may exert a measurable influence on CVD incidence. However, the extended period from 1991 to 2020 may obscure some short-term or transient factors that also contribute to disease patterns.

**Figure 10:**
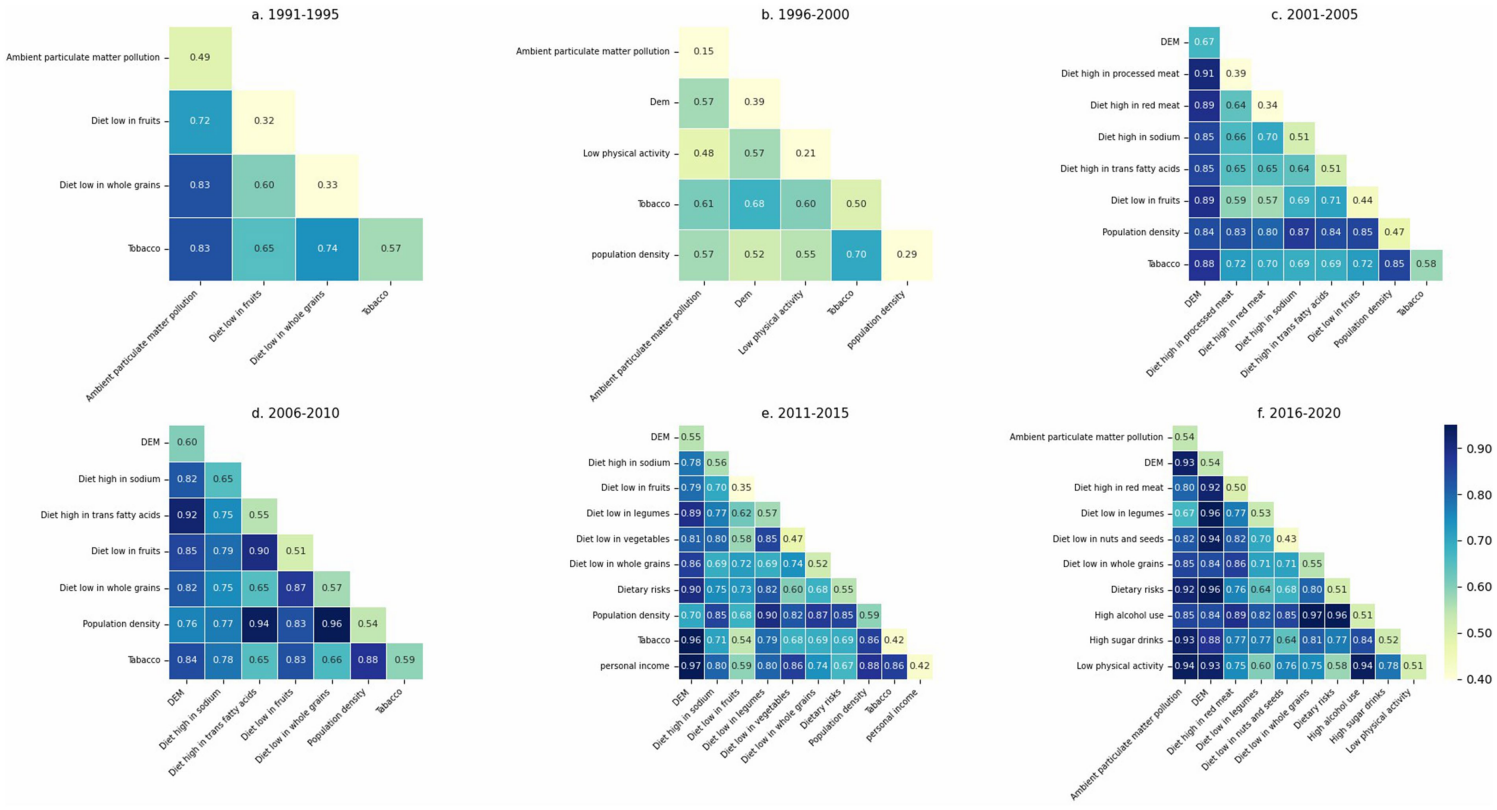
GeoDetector interaction analysis of CVD incidence in the US (1991–2020).

**Figure 11:**
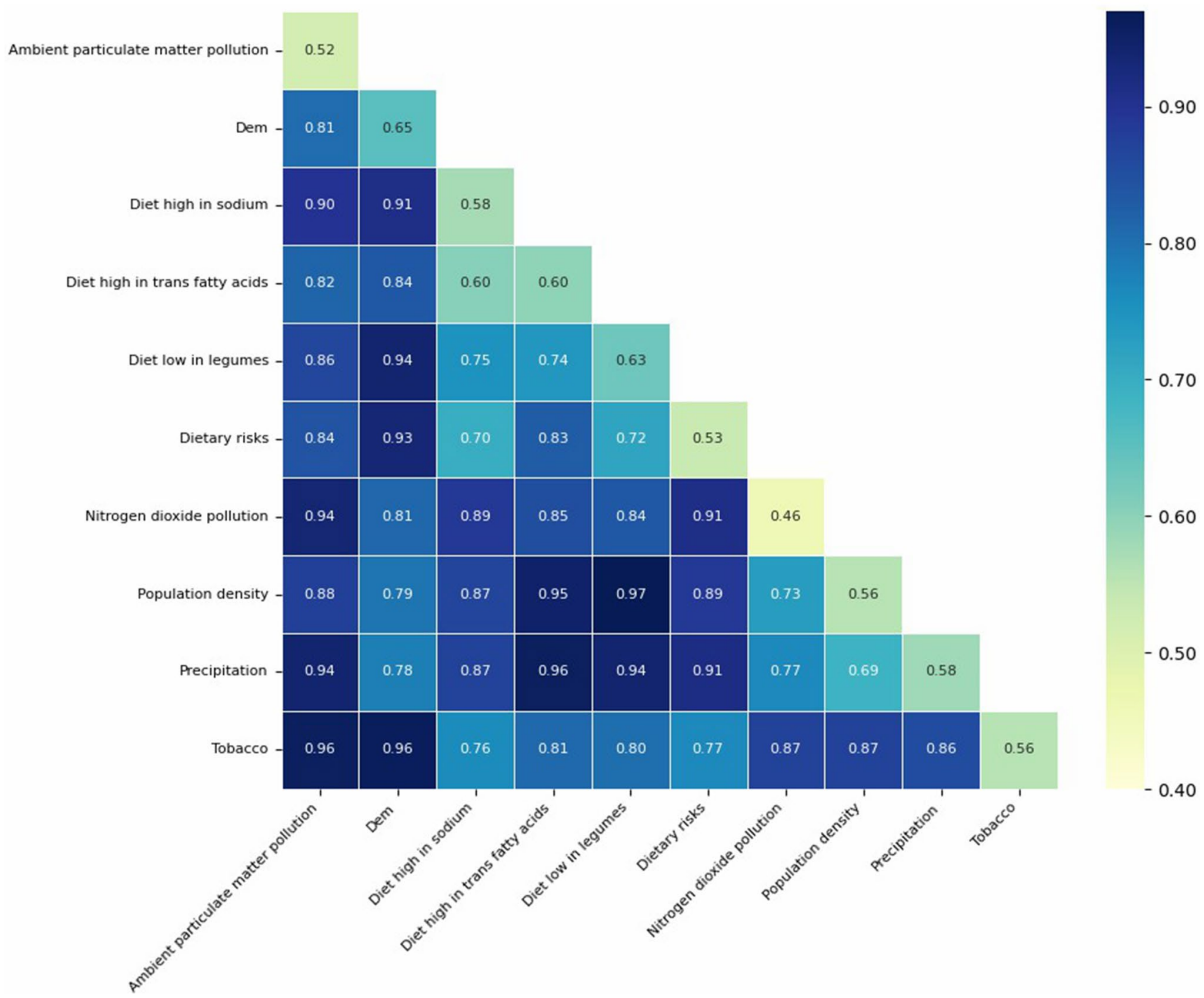
GeoDetector interaction results across the entire period.

Interaction results between key risk factors, grouped in five-year intervals, are summarized in Supplementary file 4, where each pairwise interaction type was classified according to the criteria described in Table 3. To facilitate interpretation, we also visualized the interaction strengths in Figure 10 in each five-year period. In these figures, the diagonal elements represent the individual explanatory power of each factor (q(X₁), q(X₂)), while the off-diagonal cells show the joint explanatory power of each factor pair (q(X₁, X₂)). Darker colors indicate larger values. Based on the thresholds provided in Table 3, the nature of each interaction can be inferred. In addition, Figure 11 displays the interaction results for the entire study period, providing a comprehensive overview of long-term factor interactions.

According to our analysis, whether in five-year intervals or across the entire study period, most interactions among the critical factors exhibit bilinear enhancement, suggesting synergistic but additive effects between paired variables.

#### 3.3.1 Sensitivity analysis of the GeoDetector model

To evaluate the robustness of the GeoDetector model, we conducted a sensitivity analysis by introducing a 10% random perturbation to each of the 29 independent variables. For each perturbed dataset, we recalculated the corresponding q-values and assessed the relative change compared to the original results. A smaller change indicates greater model stability.

The results of the sensitivity analysis are presented in Table 6. The analysis revealed that the average relative variation in q-values was 6.879%, which is below the 10% threshold commonly used in sensitivity assessments. This suggests that the GeoDetector model demonstrates strong robustness and reliability in the presence of minor data fluctuations, thereby supporting the credibility of the factor detection results.

**Table 6:** The results of the sensitivity analysis.

Variable	∆q/q
population_density	0.0268
Dem	0.0001
temperature	0.0647
precipitation/mm	0.0059
NDVI	0.0153
SDI	0.1572
Ambient ozone pollution	0.2022
Ambient particulate matter pollution	0.1176
Household air pollution from solid fuels	0.0442
Nitrogen dioxide pollution	0.0860
GDP	0.1353
personal income	0.2436
Diet Low in Omega-6 Polyunsaturated Fatty Acids	0.0420
Diet high in processed meat	0.0431
Diet high in red meat	0.0175
Diet high in sodium	0.0603
Diet high in sugar-sweetened beverages	0.0054
Diet high in trans fatty acids	0.0027
Diet low in fiber	0.0697
Diet low in fruits	0.0717
Diet low in legumes	0.0689
Diet low in nuts and seeds	0.0463
Diet low in seafood omega-3 fatty acids	0.0785
Diet low in vegetables	0.0496
Diet low in whole grains	0.0267
Dietary risks	0.1015
High alcohol use	0.1591
Low physical activity	0.0309
Tobacco	0.0222

### 3.4 Analysis of key influencing factors on CVD incidence using GTNNWR model

#### 3.4.1 Quantitative assessment of GTNNWR performance

As illustrated in Table 7, the GTNNWR model demonstrates excellent fitting capability and robust generalization in predicting CVD incidence. It achieves a training R^2^ of 0.9054, indicating that 90.54% of the variance in CVD incidence can be explained by the input variables on the training set. The validation R^2^ of 0.9133 confirms strong predictive performance on unseen data, with no signs of overfitting. In terms of absolute error, the model yields a Root Mean Squared Error (RMSE) of 14.41 and a Mean Absolute Error (MAE) of 10.10, suggesting that the predictions closely match the actual values. The Mean Bias Error (MBE) of 1.69 implies minimal systematic over- or underestimation, further supporting the model’s accuracy. The normalized metrics—Normalized RMSE (NRMSE) of 0.0153 and Normalized MAE (NMAE) of 0.0107—are both below 2%, which highlights the model’s robust relative performance across different spatial and temporal scales. Additionally, the NMSE loss values of 0.0022 on the training set and 0.0019 on the validation set indicate extremely low error during the learning process, reflecting the model’s strong optimization and convergence behavior. In summary, the GTNNWR model performs with high accuracy, low bias, and excellent stability in capturing the spatiotemporal non-stationarity of CVD incidence. These results suggest it is a reliable tool for dynamically identifying and modeling region-specific public health risk factors. For clarity, definitions of all quantitative metrics related to GTNNWR performance are provided in Supplementary file 1.

**Table 7:** GTNNWR model evaluation metrics.

Metric	Value
Train NMSE Loss	0.0022
Train R^2^	0.9054
Valid NMSE Loss	0.0019
Valid R^2^	0.9133
RMSE	14.4063
MAE	10.1049
MBE	1.6899
NRMSE	0.0153
NMAE	0.0107

#### 3.4.2 Key influencing factors identified by GTNNWR

**Figure 12:**
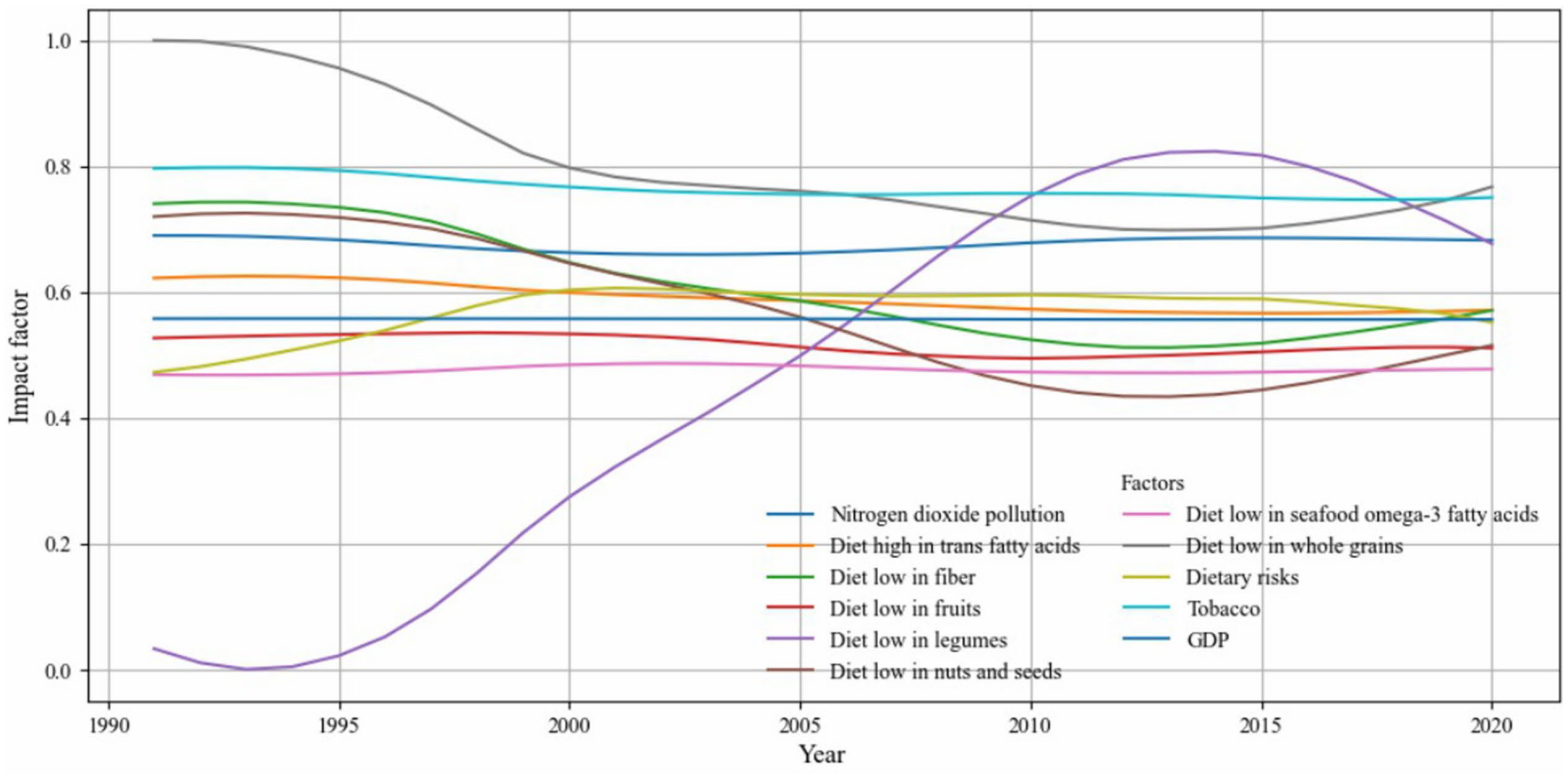
GTNNWR results of selected influencing factors.

Figure 12 highlights a subset of influencing factors that showed either consistently strong associations or substantial temporal variation in their impact on CVD incidence. Dietary risk factors such as low intake of whole grains, legumes, and fruits, as well as diet in high sodium, were among the most influential contributors. Tobacco use and nitrogen dioxide pollution also exhibited a consistently high impact across the entire study period. Notably, the influence of legume intake increased markedly after 2005, while the explanatory power of whole grain intake gradually declined after 1990. In contrast, GDP maintained a consistently low impact throughout. These trends underscore the importance of diet and behavioral factors in shaping CVD risk and reflect the model’s ability to capture nuanced, time-sensitive patterns in disease determinants. To ensure transparency, we have included the full set of visualizations covering all influencing factors in Supplementary file 5.

## 4 Discussion

CVD represents a pressing public health challenge, with increasing research dedicated to uncovering its risk factors. Traditional analytical approaches often focus on single variables or constrained geographic regions, limiting a comprehensive understanding of CVD’s multifactorial and spatially diverse nature. Unlike conventional models, GeoDetector is unaffected by multicollinearity among multiple variables (22). Instead, it analyzes the combined effects of various factors on CVD through spatial stratified heterogeneity and allows for analysis across different periods. Our findings underscore critical influences on CVD incidence, including population density, particulate pollution, and dietary patterns. This study offers a valuable framework for targeted and effective public health interventions by pinpointing high-risk areas and significant contributing factors.

Previous studies have extensively examined CVD is influenced by a combination of multiple factors. Pope et al. established a strong association between long-term exposure to air pollutants, especially PM2.5 and NO₂, and increased CVD mortality (23, 24). Franklin et al. clarified three specific mechanisms linking particulate pollution to CVD (25). Additionally, diet high in high, diet low in whole grain, and diet low in fruit have been highlighted as significant contributors to diet-related mortality and disability-adjusted life years (DALYs) (26). Our study revealed patterns that are consistent with previous findings while also providing new insights through GeoDetector analysis. Although smoking has traditionally been a prominent CVD risk factor, our results showed a decreased influence from 2016 to 2020, potentially reflecting the effectiveness of tobacco control measures (27). Furthermore, red and processed meat consumption was associated with higher CVD risk, suggesting that substituting these with plant-based foods or dairy could reduce this risk (28, 29).

The findings of our study, as revealed by GeoDetector, indicate that regional per capita GDP showed no statistically significant influence on the incidence of CVD (*p* > 0.05). This may be explained by the economic context of the US. Spiteri and von Brockdorff (2019) proposed an n-shaped relationship between economic growth and cardiovascular mortality, with mortality increasing during early economic development and declining after reaching a peak income threshold—estimated between $14,819 and $20,447 per capita (30). As the US far exceeds this threshold, additional economic growth at the macro level may not directly translate to reductions in CVD burden. It is important to note that while per capita GDP reflects the overall economic output of a region, it does not capture disparities in individual living standards. In contrast, personal income provides a more accurate representation of individuals’ access to resources such as nutritious food, clean housing, and healthcare. Biggs et al. (2010) found that the relationship between income and health within a country is heavily influenced by inequality and poverty (31). When inequality and poverty decrease, the positive effect of rising personal income on health outcomes becomes more pronounced—likely due to improved access to healthcare services. These insights underscore the importance of focusing interventions on low-income and marginalized populations, even in high-GDP settings.

While temperature and precipitation did not significantly affect CVD in five-year intervals, existing research suggests that extreme weather events, such as heat waves, could impact CVD outcomes in the short term (32, 33). This study’s large temporal and spatial scale may mask these short-term effects. Factors such as low physical activity and insufficient intake of nuts and legumes have recently emerged as significant contributors to CVD, highlighting the need for dynamic and adaptable prevention strategies that evolve with emerging risk factors.

Using Global Moran’s *I* and Getis-Ord Gi* statistics, we observed significant spatial clustering of CVD incidence across the US. Additionally, the annual Moran’s *I* index revealed that the degree of aggregation peaked in 2002, followed by a decline. While this shift likely reflects major policy interventions—such as the Clean Air Act’s reductions in ambient particulate pollution and successive AHA health-promotion goals targeting tobacco use and dietary sodium (27, 34)—it also coincides with advances in clinical care. The widespread adoption of statin therapy beginning in the late 1990s has been shown to lower population cholesterol levels and reduce coronary events (Ford et al. 2007) (35). At the same time, improvements in hypertension management and expanded use of antihypertensive medications have further driven down CVD risk (Go et al. 2014) (36). Enhanced emergency response systems and greater availability of reperfusion therapies (e.g., PCI and thrombolytics) have also contributed to reduced morbidity and mortality. While these factors may collectively explain the observed post-2002 decline, the relative contributions of each—and the potential role of additional, unexamined influences—warrant further investigation.

Our hotspot analysis identified a persistent and statistically significant concentration of CVD incidence in the Northeastern US, particularly in New York, Pennsylvania, Ohio, and West Virginia, which consistently appeared as 99% confidence hotspots from 1991 to 2020. These areas feature high population density and urbanization, often linked to greater exposure to pollutants such as nitrogen dioxide and particulate matter, as well as heightened psychosocial stress—established risk factors for CVD (23, 24, 37, 38). Socioeconomic disparities, including income inequality and housing instability, may further limit access to healthy food, healthcare, and safe environments, contributing to chronic inflammation and endothelial dysfunction (38). While the spatial pattern remained largely stable, fluctuations were noted: Washington was a cold spot during 1991–2000 but not afterward, and Tennessee and Arkansas showed hotspot characteristics between 1996 and 2005. These changes suggest that short-term policy or behavioral shifts may temporarily modulate CVD risk.

The results between GeoDetector and GTNNWR are broadly consistent. The low intake of whole grains and tobacco has been identified as a significant factor influencing CVD. Dietary factors have also become increasingly crucial in CVD incidence, and GDP is insignificant in either model. The influence of low legume intake has shown substantial variation, transitioning from a minor impact on CVD incidence to becoming the primary influencing factor between 2010 and 2015 before declining again. This fluctuation may be related to recommendations and policies from the US Food and Drug Administration (FDA). In 1999, the FDA approved a health claim linking soy protein to reduced coronary heart disease (CHD) risk based on significant scientific consensus. However, in 2007, the FDA announced plans to re-evaluate the evidence for this claim, and in 2017, citing insufficient scientific consensus, proposed revoking it (39). In most epidemiologic studies, dietary intake is merely an estimate or subjective value. Studies that do not directly measure the concentration or amount of this variable in blood or specific tissues or do not explicitly exclude the effects of other caloric substitutes have results that warrant careful consideration, as they may introduce misclassification. Additionally, many dietary factors only influence CVD when intake reaches a certain threshold, below which socioeconomic or other factors may introduce bias (39, 40). This study’s data, sourced from the GBD database, may also face similar limitations, highlighting the need for more precise and detailed original data to evaluate soy’s impact on CVD better.

Despite their overall consistency, slight differences were observed between the results of the two models. These differences may be attributed to the models’ distinct analytical approaches and temporal characteristics. The GTNNWR model is adept at capturing localized spatiotemporal effects and identifying spatial variation, while GeoDetector excels in assessing the spatial explanatory power of variables through variance analysis, particularly for factors with spatial heterogeneity. Additionally, temporal misalignment between the models could play a role, as some factors, such as prolonged exposure to certain conditions, require time to impact CVD significantly. In contrast, others, like short-term elevated temperatures or air pollution, can have immediate effects (33, 41).

## 5 Limitations

While this study provides valuable insights into the spatial–temporal heterogeneity and driving factors of CVD incidence, several limitations must be acknowledged.

First, limitations in data granularity and collection procedures may have influenced our findings. Our analysis relies on state-level data, which may overlook important variations at finer geographic scales—such as counties, cities, or neighborhoods—thereby potentially masking localized patterns in CVD incidence. This highlights the need for future research to incorporate more granular data, such as county-level health records or electronic medical data, to enable more targeted disease prevention efforts. Additionally, given the scale and complexity of GBD data collection and the involvement of numerous contributors, some degree of epidemiological uncertainty may arise from underlying assumptions or inconsistencies in data reporting.

Second, methodological limitations should be considered. The GeoDetector model requires data discretization, where variables are grouped into categories before analysis. While this approach helps assess factor influence, it may introduce classification bias, as different discretization methods can yield slightly different results. Although we applied multiple classification techniques to minimize this effect, the inherent variability remains. Similarly, the GTNNWR model, while effective in capturing spatial–temporal dependencies, requires careful parameter tuning to avoid overfitting or underfitting, which may affect the generalizability of results. Future studies could integrate alternative machine learning models, such as random forest or deep learning, to enhance robustness and predictive accuracy.

Third, the absence of certain health and socioeconomic indicators may limit the explanatory power of our models. While this study includes a diverse range of environmental, behavioral, and demographic variables, the lack of individual-level clinical data, healthcare access metrics, and genetic predisposition factors could lead to an incomplete understanding of CVD risk. Future research should incorporate multilevel data sources, including individual and community-level health records, to provide a more comprehensive picture of CVD determinants.

Finally, due to the potential impact of the COVID-19 pandemic on disease surveillance and healthcare systems, we excluded 2021 data. However, to ensure continuity in our temporal segmentation (e.g., 5-year and 10-year intervals), we retained the 2020 data to preserve analytical consistency. This decision may introduce some bias, which should be considered when interpreting the results.

## 6 Conclusion

This study systematically examined the spatiotemporal distribution and driving factors of CVD incidence across the contiguous US from 1991 to 2020, integrating spatial statistical methods with advanced deep learning models. The following key conclusions were drawn.

(1) Persistent Spatial Clustering of CVD Incidence: Global Moran’s I results consistently indicated statistically significant spatial clustering of CVD incidence across the study period, with Z-scores exceeding 2.58 in most years (*p* < 0.01). The Getis-Ord Gi* analysis further revealed persistent hot spots of high CVD incidence in the northeastern and Appalachian states, such as Ohio, West Virginia, and Pennsylvania, while cold spots were concentrated in the western and northern regions including Colorado and North Dakota. These patterns remained stable across six consecutive five-year intervals, suggesting long-term structural disparities in health outcomes. This persistent clustering underscores the necessity of regionally tailored public health interventions and continued surveillance in high-burden areas.
(2) GeoDetector analysis identified several key drivers of CVD incidence with consistently high explanatory power across time, including population density, ambient particulate matter pollution, poor dietary habits (e.g., low intake of fruits and whole grains, high sodium), and smoking. These factors showed significant q-values (*p* < 0.05), highlighting the influence of behavioral and environmental risks.
(3) Improved Modeling of Spatiotemporal Complexity: By incorporating the GTNNWR, this study advanced the modeling of non-stationary and nonlinear relationships in space and time. Unlike traditional regression models, GTNNWR dynamically assigned spatiotemporal weights, enabling more nuanced assessments of variable importance across regions and years. The model corroborated the influence of key drivers identified by GeoDetector while further revealing that their effects were not uniformly distributed. For example, dietary risks had stronger explanatory power in the Southeast, while pollution-related variables were more influential in industrialized regions. These results demonstrate the value of deep learning in enhancing the interpretability and spatial resolution of chronic disease modeling.
(4) Implications for Precision Public Health: The integration of spatial analysis and machine learning provides a robust framework for evidence-based policymaking. The identification of stable hot spots—particularly in states such as New York, Kentucky, and Louisiana—enables public health agencies to more effectively prioritize resource allocation, implement targeted screening programs, and design behavioral interventions tailored to the specific risk profiles of these high-burden regions. Furthermore, the detection of nonlinear interactions supports the design of multidimensional policies that account for synergistic risk exposure—such as combining air quality improvement with dietary education campaigns. This approach also contributes to long-term health equity by revealing regions where entrenched disadvantages continue to drive disease burden, thereby informing structural and policy-level responses beyond individual behavior change.

Together, these results demonstrate that spatial heterogeneity in CVD incidence remains a pressing public health concern. By combining interpretable spatial statistical tools and advanced neural network models, this study offers a transferable analytical framework for chronic disease surveillance and geographically adaptive intervention planning.

## Data Availability

The original contributions presented in the study are included in the article/Supplementary material, further inquiries can be directed to the corresponding authors.
