# Supplementary figures and images for: Spatio-temporal distribution of influencing factors of cardiovascular disease in the United States
Source: Front Public Health. 2025 Sep 11;13:1649851. doi: 10.3389/fpubh.2025.1649851 (PMC12460323; doi:10.3389/fpubh.2025.1649851)

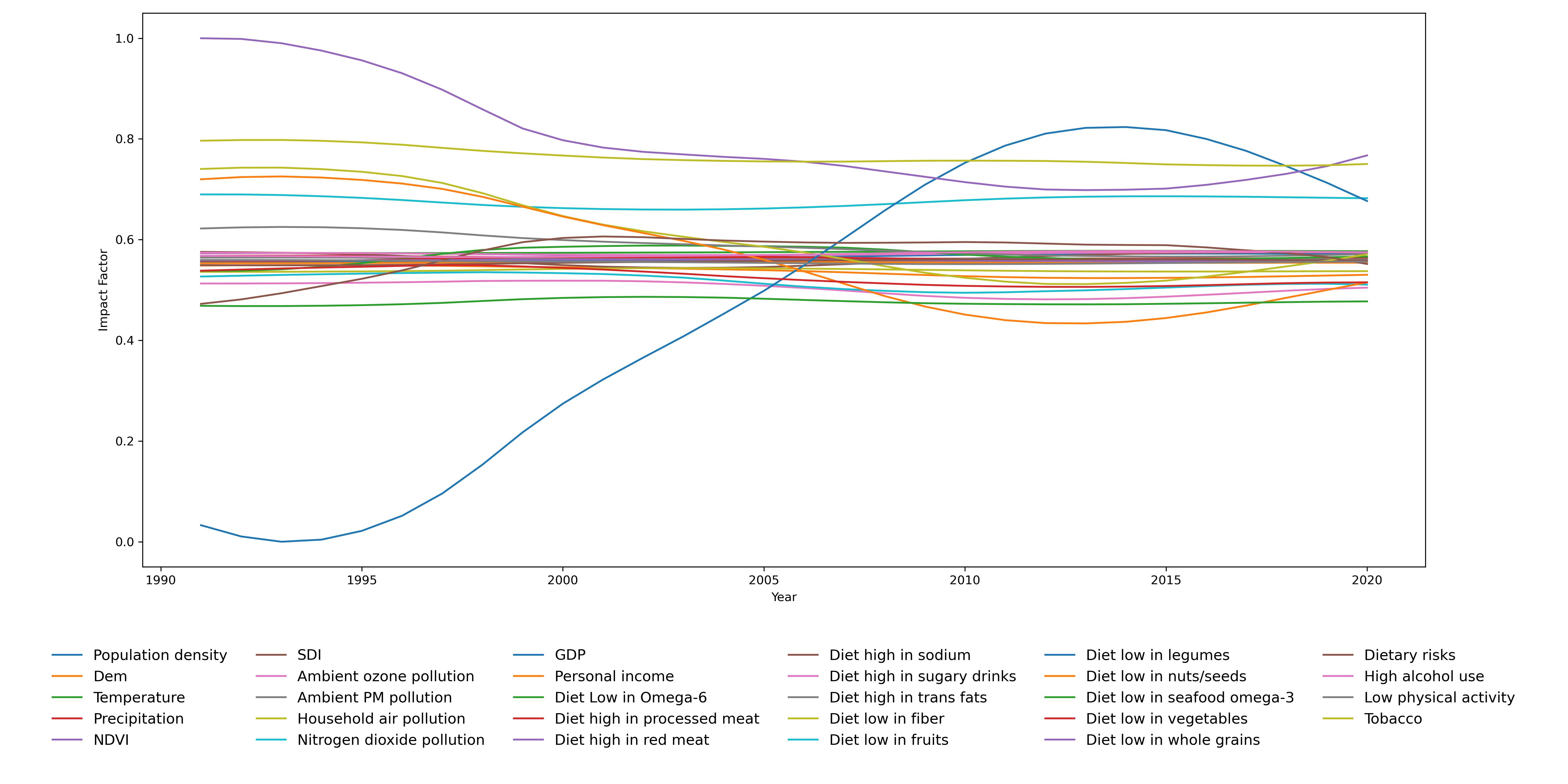

Supplement: Supplementary file 1 [file Image_1.JPEG]
