# Supplementary material for: Spatio-temporal distribution of influencing factors of cardiovascular disease in the United States
Source: Front Public Health. 2025 Sep 11;13:1649851. doi: 10.3389/fpubh.2025.1649851 (PMC12460323; doi:10.3389/fpubh.2025.1649851)
Supplement: Supplementary file 2 [file Table_1.DOCX]

**Definition of influencing factors**

1. **Ambient ozone pollution:** Ambient ozone pollution is defined as the highest seasonal (six-month) average of eight-hour daily maximum ozone concentrations. The TMREL is a uniform distribution between 29·1 and 35·7 parts per billion (ppb).
2. **Ambient particulate matter pollution:** Ambient particulate matter pollution is the population-weighted annual average mass concentration of particles with an aerodynamic diameter less than 2·5 micrometres in a cubic metre of air. The TMREL is a uniform distribution between 2·4 and 5·9 µg/m3 .
3. **Household air pollution from solid fuels:** Household air pollution includes exposure to particulate matter less than 2·5 μm in diameter (PM₂.₅) due to the use of solid fuels for cooking.
4. **Nitrogen dioxide pollution:** Exposure to nitrogen dioxide (NO_2_ ) pollution is defined as the population-weighted annual average ambient concentration of NO_2_ gas measured in parts per billion (ppb).
5. **GDP:** all references to GDP refer specifically to regional per capita Gross Domestic Product (GDP), which reflects the average economic output per person in a given state, rather than total GDP.
6. **Personal income:** Personal income is the income that is received by persons from all sources. It includes income received from participation in production as well as from government and business transfer payments.
7. **Diet Low in Omega-6 Polyunsaturated Fatty Acids:** Diet low in omega-6 polyunsaturated fatty acids is defined as average daily consumption (in % daily energy) of less than 9–10% total energy intake from omega-6, specifically linoleic acid, γ-linolenic acid, eicosadienoic acid, dihomo-γ-linolenic acid, and arachidonic acid.
8. **Diet high in processed meat:** Diet high in processed meat is defined as any intake (in grams per day) of meat preserved by smoking, curing, salting, or addition of chemical preservatives
9. **Diet high in red meat:** Diet high in red meat is defined as intake above an average of 0 grams per day (95% UI 0–200) of unprocessed red meat. Unprocessed red meat includes pork and bovine meats such as beef, lamb, and goat, but excludes all processed meats, poultry, fish, and eggs.
10. **Diet high in sodium:** Diet high in sodium is defined as average 24-hour urinary sodium excretion greater than 1–5 grams per day.
11. **Diet high in sugar-sweetened beverages:** Diet high in sugar-sweetened beverages is defined as any intake (in grams per day) of beverages with ≥50 kcal per 226·8 gram serving, including carbonated beverages, sodas, energy drinks, and fruit drinks, but excluding 100% fruit and vegetable juices.
12. **Diet high in trans fatty acids:** Diet high in trans fatty acids is defined as intake greater than 0–1·1% daily energy of trans fat from all sources, mainly partially hydrogenated vegetable oils and ruminant products
13. **Diet low in fiber**：Diet low in fibre is defined as average daily consumption (in grams per day) of less than 22–25 grams of fibre from all sources including fruits, vegetables, grains, legumes, and pulses
14. **Diet low in fruits:** Diet low in fruit is defined as average daily consumption (in grams per day) of less than 340–350 grams of fruit including fresh, frozen, cooked, canned, or dried fruit, excluding fruit juices and salted or pickled fruits.
15. **Diet low in legumes:** Diet low in legumes is defined as average daily consumption (in grams per day) of less than of 100–110 grams of legumes and pulses, including fresh, frozen, cooked, canned, or dried legumes.
16. **Diet low in nuts and seeds:** Diet low in whole grains is defined as average daily consumption (in grams per day) of less than 160–210 grams of whole grains (bran, germ, and endosperm in their natural proportion) from breakfast cereals, bread, rice, pasta, biscuits, muffins, tortillas, pancakes, and other sources.
17. **Diet low in seafood omega-3 fatty acids:** Diet low in seafood omega-3 fatty acids is defined as average daily consumption (in milligrams per day) of less than 470–660 milligrams of eicosapentaenoic acid (EPA) and docosahexaenoic acid (DHA) from seafood sources.
18. **Diet low in vegetables:** Diet low in vegetables is defined as average consumption (in grams per day) of less than 280–320 g of vegetables, including fresh, frozen, cooked, canned, or dried vegetables and excluding legumes, salted or pickled vegetables, juices, nuts and seeds, and starchy vegetables (eg, potatoes).
19. **Diet low in whole grains:** Diet low in whole grains is defined as average daily consumption (in grams per day) of less than 160–210 grams of whole grains (bran, germ, and endosperm in their natural proportion) from breakfast cereals, bread, rice, pasta, biscuits, muffins, tortillas, pancakes, and other sources.
20. **Dietary risks:** Dietary risks are an aggregate risk factor for all of the GBD dietary risks, including diet low in whole grains, fruit, fibre, legumes, nuts and seeds, seafood omega-3 fatty acids, omega-6 polyunsaturated fatty acids, vegetables, milk, and calcium; and diet high in sodium, trans fatty acids, red meat, processed meat, and sugar-sweetened beverages.
21. **High alcohol use:** Alcohol consumption in excess of the region-, age-, sex-, and year-specific theoretical minimum risk exposure level (TMREL). Current drinkers are defined as individuals consuming at least one alcoholic beverage in the past year. We estimate the level of alcohol exposure for current drinkers with the reported average grams of pure alcohol consumed per day (g/day).
22. **Low physical activity:** Low physical activity was measured in total metabolic equivalent (MET)-minutes per week and was defined as objectively measured, average weekly physical activity (at work, home, transport-related, and recreational) of less than 3600–4400 MET-minutes per week.
23. **Tobacco:** Current chewing tobacco use is defined as current daily or occasional use of chewing tobacco, including local products such as betel quid with tobacco.

    **Definitions of Evaluation Metrics for the GTNNWR Model**
24. **Normalized Mean Squared Error (NMSE):** NMSE is the mean squared error divided by the variance of the observed values. It measures the average squared deviation between predicted and actual values, normalized to account for data variability. Lower NMSE values indicate better model performance.
25. **R-squared (R²):** R² quantifies the proportion of variance in the dependent variable that is predictable from the independent variables. A higher R² indicates better goodness-of-fit, with a value of 1 representing perfect prediction.
26. **Root Mean Squared Error (RMSE):** RMSE is the square root of the mean squared error. It provides a measure of the typical magnitude of prediction errors in the same units as the dependent variable. Lower RMSE indicates higher predictive accuracy.
27. **Mean Absolute Error (MAE):** MAE is the average of the absolute differences between predicted and actual values. It provides a straightforward measure of prediction accuracy without penalizing large errors more heavily.
28. **Mean Bias Error (MBE):** MBE measures the average bias in predictions by calculating the mean difference between predicted and observed values. Positive values indicate overestimation, while negative values indicate underestimation.
29. **Normalized Root Mean Squared Error (NRMSE):** NRMSE is RMSE divided by the mean of observed values. It expresses RMSE as a proportion of the observed mean, allowing for cross-variable or cross-model comparisons.
30. **Normalized Mean Absolute Error (NMAE):** NMAE is MAE divided by the mean of observed values. Like NRMSE, it enables relative assessment of model performance across different scales or datasets.
